# Spatial and Short-Temporal Variability of δ^13^C and δ^15^N and Water-Use Efficiency in Pine Needles of the Three Forests Along the Most Industrialized Part of Poland

**DOI:** 10.1007/s11270-015-2623-z

**Published:** 2015-10-05

**Authors:** Barbara M. Sensuła

**Affiliations:** Institute of Physics-Center for Science and Education, Silesian University of Technology, Konarskiego 22B, 44-100 Gliwice, Poland

**Keywords:** Stables isotopes, Carbon, Nitrogen, WUE, Atmospheric pollution, Pine needles

## Abstract

In this study, stable carbon and nitrogen isotope ratios in the samples of pine needles collected in 2013 and 2014 from heavily urbanized area in close proximity to point-source pollution emitters, such as a heat and power plant, nitrogen plant, and steelworks in Silesia (Poland), were analyzed as bio-indicators of contemporary environmental changes. The carbon isotope discrimination has been proposed as a method for evaluating water-use efficiency. The measurement of carbon and nitrogen isotopes was carried out using the continuous flow isotope ratio mass spectrometer. The isotope ratio mass spectrometer allows the precise measurement of mixtures of naturally occurring isotopes. The δ^15^N values were calibrated relative to the NO-3 and USGS34 international standards, whereas the δ^13^C values were calibrated relative to the C-3 and C-5 international standards. The strong year-to-year correlations between the δ^13^C in different sampling sites, and also the inter-annual correlation of δ^15^N values in the pine needles at each of the investigated sampling sites confirm that the measured δ^13^C and δ^15^N and also intrinsic water-use efficiency (iWUE) trends are representative of the sampling site. Diffuse air pollution caused the variation in δ ^13^C, δ^15^N, and iWUE dependent on type of emitter, the localization in the space (distance and direction) from factories and some local effect of other human activities. The complex short-term variation analysis can be helpful to distinguish isotopic fractionation, which is not an effect explainable by climatic conditions but by the anthropogenic effect. Between 2012 and 2014, an increase in iWUE is observed at leaf level.

## Introduction

The reconstruction of atmospheric pollution is essential in order to evaluate the impact of environmental regulations on the forest ecosystem and human health. To reconstruct the ecosystem changes, caused by climate changes and human activities, different natural archives that present long-term data are used, for example: lake sediments, peat, and trees (Sensuła et al. 2006; Fiałkiewicz-Kozieł et al. 2014). There is a lack of analysis concerning short-term changes in ecosystem showing the gradient of the changes in the area near emitters of pollutants in the contemporary environment. This information can be crucial for the analysis of long-term data, especially during choosing the references and representative sampling sites, where the climatic signals recorded in tree rings can be masked by the anthropogenic effect signal which can vary in time and space. Nevertheless, there is lack of contemporary spatiotemporal analysis of the current changes in environment. The analysis concerning short-term changes in ecosystem which show the gradient of the changes in the area near emitters of pollutants in the contemporary environment can be crucial also, for example, for reclamation of degraded landscapes in the post-industrial period of time. Most of the modernization in different plants and the industrial sector in Eastern Europe are connected with EU legislation and the implementation of restrictive governmental regulations on emissions. In Poland, similarly as in most countries all over the world, the systematic long-term monitoring of air pollutants is generally restricted to rural point-source regions in urban areas.

Several studies have been successful to infer long-term trends of point-source air pollution involving different types of industrial production such as, for example, power plants, chemical plants, and copper and metal smelters (Szychowska-Krąpiec and Wiśniowski 1996; Wilczynski 2006; Wagner and Wagner 2006; Savard 2010; Malik et al. 2012; Sensuła et al. 2011), and only a few studies have demonstrated the spatiotemporal analysis of ecosystem changes and air pollutant distribution in the space within an industrial area. Usually, one master chronology for the investigated population within the sampling site is created, and some information about the specificity of the area can be hidden by averaging. While, a detailed analysis of diffuse air pollution signal recorded in the stable isotope composition of trees can show more detailed and contrasted responses to environmental changes (e.g., Farquhar and Lloyd 1993; McCarroll and Loader 2004; Saurer et al. 2004; McCarroll et al. 2009; Choi et al. 2005; Sensuła and Pazdur 2013a; Pazdur et al. 2007; Saurer et al. 2014). Isotopic fractionation refers to any process that changes the relative abundances of stable isotopes of an element. Only few studies use different stable isotopic composition in the leaves as bio-indicators (e.g., Sensuła et al. 2015; Ehleringer 1990; Gebauer et al. 1994) in the analysis of diffuse atmospheric pollution). It was possible to observe a spatiotemporal fractionation of carbon stable isotopes in the needles of pine in two consecutive years in the area near heat plant (Sensuła et al. 2015). The combination of several independent indicators constitutes a powerful tool as an example in environmental, ecological, or dendrochronological research. The identification and quantification of sources of carbon and nitrogen, and their temporal and spatial variability on both global and regional scales is a prerequisite for a better understanding of the dynamics of the carbon and nitrogen cycle and its response to the ever-increasing human impact (IPCC 2001; Levin et al. 2010). According to report of the European Environment Agency (2013), exposure to air pollution has also been linked to low birth weights in babies and has also been linked to asthma, heart disease, and kidney damage.

The stable nitrogen and carbon isotopes are used as indicators widely applied in environmental and ecological fields (e.g., Emmett et al. 1998). The anthropogenic N emission into the atmosphere has increased N deposition dramatically over the last century, resulting from increased fossil-fuel combustion and fertilizer (Erisman and de Vries 2000). Whereas, the observed anthropogenic impact the global carbon cycle, mainly related to fossil-fuel and biomass burning, land-use changes, and various industrial activities (Martin et al. 1988; Marland et al. 2008) caused changes in the isotopic composition of carbon in the atmosphere and also in the biosphere. Human alterations of the carbon and nitrogen cycle have influenced the dynamics, biodiversity, and functioning of many ecosystems and ecological processes (Vitousek et al. 1997). Some of the pollutants are restricted to small, near-source regions, whereas the others are distributed over much larger areas. Abrupt changes in environmental conditions, such as an increase in air pollution, land use, and climate changes can be responsible for the occurrence of abrupt growth reductions or missing rings (Schweingruber 1986, 1996) and also for differences in carbon, oxygen, and nitrogen isotopic fractionation (Farquhar and Lloyd 1993; Saurer et al. 2004; McCarroll et al. 2009; Guerrieri et al. 2011; Choi et al. 2005; Sensuła and Pazdur 2013b; Pazdur et al. 2013).

Carbon isotope fractionation is highly correlated with plant water-use efficiency (Farquhar and Lloyd 1993), which is the ratio of photosynthetic carbon assimilation to transpiration, because water is commonly the most limiting environmental factor for tree growth. Water may be especially limiting in urban environments where limited soil volumes, soil compaction, and elevated temperatures can combine to increase tree moisture stress (e.g., Cregg 1993). Spatial variability and temporal trends in water-use efficiency of European forest till 2000 ad has been studied in several tree species. The magnitude and spatial patterns of water fluxes passing through stomata in natural forests remain highly uncertain (Saurer et al. 2014). It is reported that the relationship between δ^13^C and plant water availability is not linear, showing a saturation trend as water availability increases (Warren et al. 2001), and over a global survey of δ^13^C values on conifers, δ^13^C reached an asymptotic value once there is no water deficit, namely when the ratio between precipitation and evapotranspirative demand equalled unity. The reason for that general trend is that the main factor relating δ^13^C with water inputs is stomatal conductance, which is expected to reach its maximum in non-stressed plants. Under optimum water availability, no further increments in stomatal conductance, and thus on δ^13^C, would be expected (Lambers et al. 1998).

To analyze the ecosystem changes, caused by human activities, usual tree-ring series that present long-term data are used (e.g., Battipaglia et al. 2013; Saurer et al. 2014), but there is still a lack of analysis of contemporary forest in the most industrialized part of Europe—the southern part of Poland, where reclamation of degraded landscapes takes a place in the post-industrial period of time.

This article presents investigations of nitrogen and carbon isotopic composition in samples of pine (*Pinus silvestris* L.) needles collected in the Silesia area, exposed to anthropogenic stress typical for heavily urbanized areas. The areas were in close proximity to the heat and power plant in Łaziska (LA), the nitrogen plant in Kędzierzyn-Kozle (KK), and the steelworks in Dabrowa Gornicza (HK). The short-term isotopic pattern of air pollution diffuse was analyzed taking the dominant wind direction and distance from the industrial company into account. The objectives of this study were to determine if there are significant differences of δ^13^C and δ^15^N among different distance from different large emitter pollution sources. The second goal of these studies was to determine a spatial variability and temporal trends in intrinsic water-use efficiency in contemporary forests located across the most industrialized and important part of Poland, where the large parts of the population do not live in a healthy environment, according to current standards.

## Materials and Methods

### Site Description

**Fig. 1:**
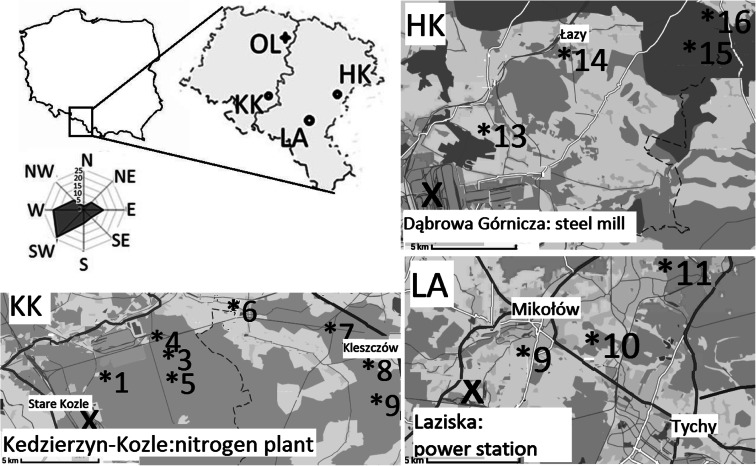
Sampling sites near the nitrogen power plant in Kędzierzyn Koźle (*KK*), combined heat and power plant in Łaziska (*LA*), steel mill in Dąbrowa Górnicza (*HK*), and in a comparative site in Olesno (*OL*)

The three selected sites (Fig. 1; Table 1) were located along the southern part of Poland, in the region which is one of the most polluted regions in Poland and in Europe, highly industrialized over the years and highly populated.

**Table 1:** Sampling stand and site near the nitrogen plant in Kędzierzyn Koźle (KK), combined heat and power plant in Łaziska (LA), steel mill in Dąbrowa Górnicza (HK), and in comparative site in Olesno (OL)

Lab code			Distance from plants (km)	Geographical coordinates
Number	Stand	Sampling site		
1	KK	KKA1/1	1	50° 19′ 15.2″ N; 18° 15′ 25.6″ E
2	KK	KKA_5/4	4	50° 17′ 17.6″ N; 18° 19′ 29″ E
3	KK	KKC_5/6	6	50° 20′ 15.7″ N; 18° 19′ 52.5″ E
4	KK	KKBL_10/6	6	50° 21′ 0.08″ N; 18° 18′ 57.7″ E
5	KK	KK_10/6	6	50° 20′ 3.6″ N; 18° 20′ 29.9″ E
6	KK	KKN_10/11,5	11.5	50° 22′ 14.9″ N; 18° 23′ 39.9″ E
7	KK	KK_15/16	16	50° 21′ 26.4″ N; 18° 28′ 14.5″ E
8	KK	ZKK_20b/17	17	50° 19′ 55.6″ N; 18° 30′ 11.8″ E
9	LA	LAW_5/3	3	50° 9′ 38.8″ N; 18° 56′ 4.4″ E
10	LA	LAM_10/7	7	50° 10′ 35.1″ N; 18° 58′ 52.8″ E
11	LA	LAP_15/11	11	50° 8′ 55.4″ N; 18° 53′ 4.9″ E
12	HK	HK_5/3	3	50° 21′ 48.5″ N; 19° 19′ 24″ E
13	HK	HKG_10/10	10	50° 24′ 58.4″ N; 19° 22′ 56.8″ E
14	HK	HK_ 20/14,5	14.5	50° 24′ 13.16″ N; 19° 28′ 56.9″ E
15	HK	HKO_15/17	17	50° 26′ 27″ N; 19° 29′ 30.6″ E
16	OL	OL	100	50° 47′ 55.3″ N; 18° 23′ 20.1″ E

The first sampling area was located near the nitrogen plant in KK, the second in to the vicinity of the heat and power plant in LA, and the third one near the steelworks in HK. All of these factories have, for a long time, been on the list of the most environmentally burdensome industries in Poland. It should be mentioned that coal has been the traditional fuel for this region and has contributed 83 % of the total fossil-fuel CO_2_ emissions in 1950. Most recently, the use of gas fuels has increased dramatically, and since 1998, emissions from gas fuels have the exceeded emissions from coal (Marland et al. 2008) According to report of the European Environment Agency (2013), in the investigated region, a significant increase of SO_2_, NO_*x*_, and CO_*x*_ emissions is an effect of industrialization of Silesia since the end of the nineteenth century up to now. Most of the modernization in those plants, similar like in industrial sector in Eastern Europe, was connected with access to EU funding in the past decades and EU legislation and the implementation of restrictive governmental regulations on emissions.

### Samples Collection

The sampling sites within three research areas were located in different distances from the source of pollution (from 1 to 20 km). Additionally, one comparative site was selected in a relatively clean environment, about 100 km NW from the emitter (Malik et al. 2012). Selected areas were sampled in 2013 (winter, 12 samples and summer, 16 samples) and 2014 (summer, 16 samples). The samples of needles collected in January 2013 were created in the previous year—in 2012. The needles collected in September 2013 were created during 2013, and the needles collected in July 2014 were also created during 2014, respectively. All the needle samples were collected on the same day, to remove the influence of changes in weather conditions, which can influence the samples. The needles were collected from the upper sun crown of a minimum of three trees per site (Table 1; Fig. 1). The needles were washed in distilled water, dried and then homogenized, cut into small pieces, and powdered with mortar. The sheaths on the needles were carefully removed prior to the division.

### Isotope Analysis

Isotopic values are reported in a standard notation as delta:

$$ \delta =\frac{R_{\mathrm{sample}}-{R}_{\mathrm{standard}}}{R_{\mathrm{standard}}} $$

where *R* represents the ratio of the heavy to light isotope in the sample and in the standard. The δ^13^C and δ^15^N results are reported in values relative to VPDB.

### Carbon Isotope Discrimination and Water-Use Efficiency

The carbon isotope discrimination is calculated as follows (Farquhar and Lloyd 1993):

$$ {\Delta}^{13}\mathrm{C}=\frac{\updelta^{13}{\mathrm{C}}_{\mathrm{air}}-{\updelta}^{13}{\mathrm{C}}_{\mathrm{plant}}}{1+\frac{\updelta^{13}{\mathrm{C}}_{\mathrm{plant}}}{1000}} $$

where ^13^C_air_ and δ^13^C_plant_ represents air and plant composition, respectively. Present δ^13^C in air is about −8 ‰, whereas typical C_3_ leaf composition becomes −29 ‰. The main factors determining δ^13^C in C_3_ plants are diffusion in the air (including the boundary layer and the stomata) and carbon fixation by the carboxylation enzyme ribulose bisphosphate carboxylase (Ferrio et al. 2003; Farquhar and Lloyd 1993; Farquhar et al. 1989).

The isotopic discrimination in photosynthesis can be described as the model of (Farquhar and Lloyd 1993):

$$ {\varDelta}^{13}\mathrm{C}=a+\left(b-a\right){C}_{\mathrm{i}}/{C}_{\mathrm{a}} $$

where *C*_i_ is inter-cellular CO_2_ concentration, *C*_a_ is ambient CO_2_ concentration, *a* (ca. 4.4 ‰) is the discrimination against ^13^CO_2_ during CO_2_ diffusion through stomata, and *b* (ca. 27 ‰) is the discrimination associated with carboxylation.

Carbon isotope discrimination (δ^13^C) is related to intrinsic water-use efficiency (iWUE) because iWUE is a measure of the amount of water loss per unit carbon gained (Saurer et al. 2004)

$$ \mathrm{iWUE}=A/{g}_{\mathrm{s}}=\left({C}_{\mathrm{a}}-{C}_{\mathrm{i}}\right)/1.6 $$

where *A* is photosynthesis net, *g* is stomatal conductance, and 1.6 is the ratio of diffusivities of water and CO_2_ in air.

The measurement of carbon and nitrogen isotopes were carried out using the elemental analyzer coupled to the continuous flow isotope ratio mass spectrometer (EA-CF-IRMS).

The stable carbon and nitrogen isotope compositions of the samples were determined using an IsoPrime elemental analyzer/continuous flow isotope ratio mass spectrometer (GV Instruments, Manchester, UK) at the Mass Spectrometry Laboratory of the Silesian University of Technology (Sensuła et al. 2011; Sensuła and Pazdur 2013a, b). The continuous flow (CF) sample introduction technique consists of a helium carrier gas that carries the analyte gas into the ion source of the IRMS. In order to determine the δ^13^C and δ^15^N values, the samples (0.15 mg for carbon and 1 mg for nitrogen) were encapsulated in a tin. The samples were combusted at a temperature of 1020 °C in elemental analyzer. The samples then passed through a trap to remove the H_2_O. In case of nitrogen isotope analysis, the samples passed also through a trap with Carbosorb to remove CO_2_. Then, the analyte gases were separated from each other and also from impurities on a packed GC column. The δ^15^N values were calibrated relative to the NO-3 and USGS34 international standards, whereas the δ13C values were calibrated relative to the C-3 and C-5 international standards. The δ^13^C and δ^15^N results are reported in values relative to VPDB. The precision on triplicates was ±0.26 ‰ (*n* = 50) for δ^13^C and ±0.5 ‰ (*n* = 45) for δ^15^N.

## Results

**Fig. 2:**
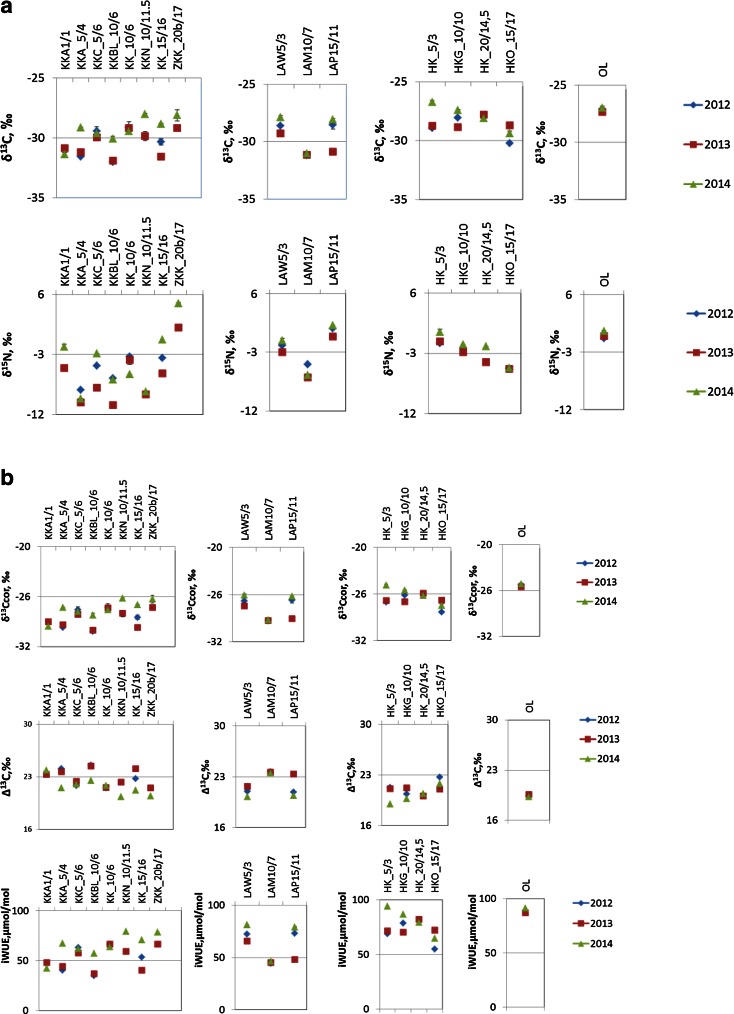
Spatial and short-temporal variability of δ^13^C and δ^15^N (**a**) and δ^13^C value corrected to Suess effect, carbon isotope discrimination, and water-use efficiency (**b**) in pine needles created in 2012–2014, collected from three industrialized forest: near the nitrogen plant in Kędzierzyn Koźle (*KK*), combined heat and power plant in Łaziska (*LA*), steelworks in Dąbrowa Górnicza (*HK*), and in comparative site in Olesno (*OL*)

The software *Statistica 10* (Statsoft Inc. 2011) was used in statistical analysis. Pattern of spatial and short-temporal variability of δ^15^N, δ^13^C, Δ^13^C, and water-use efficiency in pine needles of the three forests along the most industrialized part of Poland, in close proximity to the heat and power plant in LA, the nitrogen plant in KK and the steelworks in HK are illustrated (Fig. 2) and summarized in Table 2. Additionally, the results of δ^15^N, δ^13^C, Δ^13^C, and water-use efficiency in pine needles in one comparative (OL) site are also presented in Table 2.

**Table 2:** δ^13^C and δ^15^N, carbon isotope discrimination (Δ), and water-use efficiency values (minimum, maximum, average) measured in pine needles created in 2012, 2013, and 2014 at the area near the nitrogen plant in Kędzierzyn Koźle (KK), combined heat and power plant in Łaziska (LA), and steelworks in Dąbrowa Górnicza (HK)

	2012					2013					2014				
	Number of sampling sites	Minimum	Maximum	Mean	Standard deviation	Number of sampling sites	Minimum	Maximum	Mean	Standard deviation	Number of sampling sites	Minimum	Maximum	Mean	Standard deviation
Kedzierzyn-Kozle (KK; nitrogen plant)															
δ^13^C (‰)	5	−32.0	−29.1	−30.5	1.3	8	−31.9	−29.2	−30.5	1.1	8	−31.4	−28.0	−29.3	1.1
δ^15^N (‰)	5	−10.9	−4.7	−6.9	2.4	8	−10.4	1.8	−6.2	3.9	8	−9.4	4.7	−3.7	4.6
Δ^13^C (‰)	5	21.5	24.6	22.9	1.3	8	21.5	24.4	22.9	1.1	8	20.4	23.9	21.7	1.1
iWUE (μmol/mol)	5	35	67	52	14	8	37	67	52	12	8	42	79	65	12
Laziska (LA; heat and power plant)															
δ^13^C (‰)	3	−31.2	−28.5	−29.5	1.5	3	−31.1	−29.3	−30.4	1.0	3	−31.1	−27.9	−29.0	1.8
δ^15^N (‰)	3	−3.5	2.1	−0.5	2.9	3	−6.2	0.3	−2.7	3.3	3	−6.3	2.1	−1.5	4.3
Δ^13^C (‰)	3	20.9	23.7	21.8	1.6	3	21.7	23.6	22.9	1.1	3	20.2	23.5	21.4	1.9
iWUE (μmol/mol)	3	44	73	63	16	3	45	66	53	11	3	46	81	69	20
Huta Katowice (HK; steelworks)															
δ^13^C (‰)	3	−30.24	−28.05	−29.1	1.1	4	−28.9	−27.8	−28.5	0.50	4	−29.4	−26.7	−27.9	1.1
δ^15^N (‰)	3	−5.2	0.0	−1.9	2.9	4	−5.3	−0.6	−3.1	2.1	4	−5.2	0.2	−2.2	2.2
Δ^13^C (‰)	3	20.37	22.66	21.4	1.2	4	20.1	21.2	20.9	0.5	4	19.0	21.8	20.2	1.2
iWUE (μmol/mol)	3	54.7	78.5	67	12	4	70	82	73.9	5.5	4	65	94	81	12
Olesno (OL; comparative site)															
δ^13^C (‰)	1	−27.10				1	−27.35				1	−27.00			
δ^15^N (‰)	1	1.48				1	−0.31				1	2.25			
Δ^13^C (‰)	1	19..36				1	19.63				1	19.26			
iWUE (μmol/mol)	1	88.91				1	86.74				1	91.08			

Average value of δ^13^C and δ^15^N in all the investigated sites (total) and in comparative site in Olesno (OL)

**Fig. 3:**
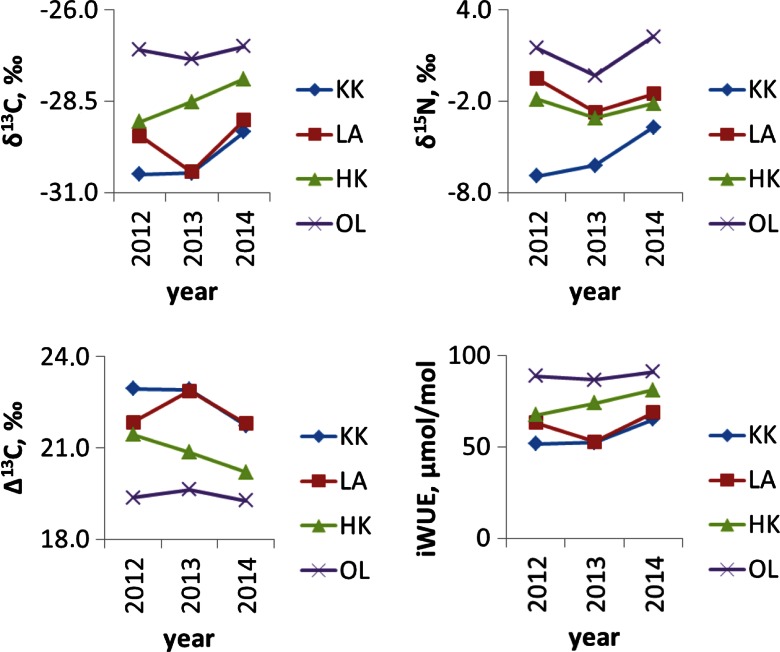
Comparison of trends of δ^15^N, δ^13^C, Δ^13^C, and intrinsic water-use efficiency (*iWUE*) means annual value in pine needles from the three industrialized forests: the area near the nitrogen plant in Kędzierzyn Koźle (*KK*), combined heat and power plant in Łaziska (*LA*), steelworks in Dąbrowa Górnicza (*HK*), and in comparative site in Olesno (*OL*)

Comparison of mean annual trends of δ^15^N, δ^13^C, Δ^13^C, and water-use efficiency in pine needles between research areas are presented in Fig. 3. Year-to-year analysis shows that between 2012 and 2014, iWUE increased in all investigated sites and a similar trend can be observed in annual tree rings across Europe (Saurer et al. 2014).

**Fig. 4:**
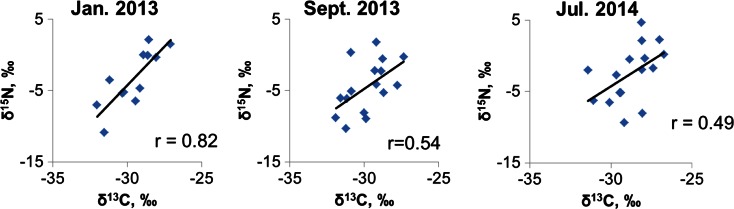
Combinations of δ^13^C and δ^15^N for pine needles from all the sampling sites, sampled in 2013 (winter, 12 samples and summer, 16 samples) and 2014 (summer, 16 samples)

The strong year-to-year correlation between the δ^13^C values in the needles of pine at each of the studied areas and sampling sites confirm that the measured δ^13^C, δ^15^N, and iWUE trends are representative of the sampling site. In addition, the δ^13^C inter-sampling site patterns of needle series are similar for all sites (*r* > 0.65, *p* < 0.05 for δ^13^C, *r* ≥ 0.84, *p* < 0.05 for δ^15^N, and *r* ≥ 0.62, *p* < 0.05 for iWUE, respectively). Additionally, the strong inter-annual correlation of the δ^15^N values at each of the studied areas and sampling sites confirm that the measured δ^15^N trends are representative of the sampling sites. Combinations of δ^13^C and δ^15^N for pine needles from all the sampling sites, sampled in 2013 (winter, 12 samples and summer, 16 samples) and 2014 (summer, 16 samples) are illustrated (Fig. 4). A significant correlation between δ^13^C and δ^15^N (*r* = 0.82) was noted between δ^13^C and δ^15^N in the pine needle samples created in 2012 and collected in the winter in 2013. It is possible that it is connected with the vegetation period that begins in Poland at the end of March or beginning of April and finishes in September. Collecting samples in July facilitates the collection of very young “fresh” needles. The sensitivity of pine on air pollution is also connected with pine physiology.

## Discussion

Atmospheric N and C deposition has affected the forest ecosystems in the Silesian region, in the southern part of Poland for a long period of time (Marland et al. 2008). In this study, the first step is to explore the potential of using δ^13^C and δ^15^N pine needles (*Pinus sylvestris* L.) to indicate the difference of plant-available C and N among the research areas and sampling sites within the areas, respectively. A detailed analysis of the results show the variation of the stable isotope composition at the sampling sites located at different distances from the plants within the investigated areas (Fig. 2). Taking into account the wind direction (Fig. 1), it is not possible to completely exclude the impact of pollution from a long-range transport. Significant differences of the annual average of δ^15^N values in the needle were observed from the relatively healthy and polluted sites. The lowest value of the annual average of δ^15^N is observed in proximity of the nitrogen plant in the KK area, whereas the highest value of the annual average of δ^15^N is observed in the site near the heat and power plant in LA. Significant differences of the needle average δ^13^C values are observed from the relatively healthy and the polluted sites. The highest value of the annual average of δ^13^C is observed in proximity to steelworks sampling site (HK), whereas the highest value of annual average of the δ^15^N is observed in the site near the heat and power plant in LA (Fig. 2). For three consecutive years, the variation of amplitude of the average value of the isotopic composition in pines collected from three polluted sites is smaller for carbon isotopes than for nitrogen isotopes in the needles created in 2012, 2013, and 2014 (Fig. 3).

### δ^15^N

This research shows that the relative importance of the different sources of N for the plant N budget can be derived at the leaf level by using the N isotope (Bukata and Kyser 2007; Siegwolf et al. 2001). Spatially inhomogeneous δ^15^N changes as a function of the type of emitters, the space around the emitters (distance and direction from emitters), wind direction, and local road-effects at all areas investigated can be noted (Figs. 1 and 2; Table 2). The gradient changes of δ^15^N from year to year can be observed. The highest fluctuation can be observed constructed in proximity with the nitrogen plant in Kędzierzyn-Koźle. In general, the obtained results showed that the distance from all the plants has an influence on the δ^15^N in the needles of pine growing in the industrialized forest and a decrease of δ^15^N with a corresponding increase in the distance could be observed. It was found that the highest values of δ^15^N at a distance of 4, 16, and 17 km from nitrogen plant (sampling sites KKA_5/4, KK_15/16, and ZKK_20b/17) were due to road pollution. Both sites (KK_15/16 and ZKK_20b/17) are located in the nearby motorway. The nitrogen isotopic ratios changes under stress due to road pollution (Saurer et al. 2004; Leonelli et al. 2012). Probably, the same effect is observed in LAP15/11 population of pine at a distance of 11 km from the heat and power plant in Łaziska. For the 2012 and 2014 period, a similar pattern of δ^15^N annual average value is observed in population of pine growing in HK, LA, and comparative site (OL). In case of pine population, KK increase of δ^15^N annual average value is noted (Fig. 3).

### δ^13^C

These studies show that the relative importance of different sources of C for the plant C budget can be derived at the leaf level by using the C isotope. The gradient changes of δ^13^ C from year to year can be observed. Spatially inhomogeneous of δ^13^C changes as a function of the type of emitters, the space around the emitters (distance and direction from emitters), wind direction, at all investigated can be noted (Figs. 1 and 2; Table 2). All δ^13^C values in the pine needles collected from the three forests along the most industrialized part of Poland, in close proximity to the heat and power plant in LA, the nitrogen plant in KK and the steelworks in HK show that the δ^13^C value of needles from all the investigated sampling sites are lower than the δ^13^C value of the needles from the comparative site (OL) selected in the relatively clean environment, about 100 km NW from the emitters. This effect of the decrease can be explained as the known effect of the decreasing δ^13^C in the air, and the biosphere is associated with the increase of CO_2_ in the atmosphere. According to the NOAA, the average global atmospheric CO_2_ concentration *has risen* from 394 ppm in 2012 to 399 ppm in 2014. The observed anthropogenic impact on the carbon cycle is mainly related to various industrial activities (Marland et al. 2008), which also caused changes in the isotopic composition of carbon in the ecosystem (Pazdur et al. 2013). Plants grown at the higher level had a more negative δ^13^C than plants grown at the lower concentration. In C_3_ plants, CO_2_ is usually limiting photosynthesis and, thus, an increase in CO_2_ results in greater photosynthetic rates. On the other hand, plants take advantage of the increased CO_2_ availability to augment water-use efficiency (i.e., the ratio between net assimilation and water transpired) by closing stomata. This reduction in stomatal conductance does not limit photosynthesis, thus δ^13^C values become more negative as CO_2_ concentration increases (Polley et al. 1993). Therefore, CO_2_ and light gradients may have additive effects within closed canopies, both contributing to leaf δ^13^C decrease with depth (Broadmeadow and Griffiths 1993). The detailed analysis of the research areas and sampling sites show that local Suess effect can be noted from year to year. The influence of SO_2_ on δ^13^C value cannot be excluded. Whereas, SO_2_ emissions can also increase the tree-ring δ^13^C values by augmenting dark respiration and changing the photosynthate allocation and partitioning (Wagner and Wagner 2006; Rinne et al. 2010).

### iWUE

Spatially, inhomogeneous iWUE changes as a function of the type of emitters, the localization of sampling sites around the emitters (distance and direction from emitters), wind direction, and local road-effects at all areas investigated can be noted (Figs. 1 and 2; Table 2). Results show a remarkable agreement regarding the spatial patterns characterized by an increasing trend in iWUE with increasing distance from industrial companies at almost all places in KK and LA sampling sites from year to year. Only in the case of HK can a decrease of iWUE with an increase of distance from steelworks can be noted. As it was mentioned above, δ^13^C values become more negative as CO_2_ concentration increases, whereas SO_*x*_ emissions can also increase the tree-ring δ^13^C values by augmenting dark respiration and changing the photosynthate allocation and partitioning (Wagner and Wagner 2006; Rinne et al. 2010). It is possible that the wide-spread iWUE increase was likely caused by a reduction in the stomatal conductance at the needles (Gagen et al. 2011). As the stomata tend to close under elevated CO_2_ concentration, this mechanism for saving water often results in an improvement of the iWUE, the water-used per unit carbon gain at the needle level. The spatial variation of iWUE patterns and trends can be explained by a variation of soil water content and air pollution, e.g., CO_2_, nitrogen, sulfur dioxide, and ozone, which also influence tree physiological properties such as photosynthesis and stomatal conductance. In this study, year-to-year analysis shows that between 2012 and 2014, iWUE increased in all investigated sites and a similar trend can be observed in annual tree rings. The magnitude and spatial patterns of water fluxes passing through stomata in natural forests remain highly uncertain (Battipaglia et al. 2013; Saurer et al. 2014). It was observed (Saurer et al. 2014; Rinne et al. 2010) that long exposure of trees to a sulfur dioxide background influenced carbon isotope signatures and iWUE remote from industrial areas; however, it was expected that through legislation concerning reduction of pollution, the influence of nitrogen and sulfur was expected to further decrease in the future relative to climate change and CO_2_ effect. In 2012, the average global atmospheric CO_2_ was ca. 394 ppm, while in 2014, the average global atmospheric CO_2_ was ca. 399 ppm (NOAA). In Poland, according to the Statistical Review of World Energy (2015), the carbon dioxide emission was ca. 329 million tons of carbon in 2012, ca. 329.1 million tons of carbon in 2013, and ca. 316.8 million tons of carbon in 2014. Local and global emission of carbon dioxide has influenced the carbon stable isotope composition of pine and iWUE The most negative δ^13^C are observed in most samples created in 2013, whereas the less negative value of δ^13^C are observed in samples collected in 2014 For the 2012 and 2014 period, a local increase of annual average iWUE is observed by ca. 6 % (KK) and by ca. 13 % (LA and HK), while in comparative site (OL), an increase of annual average iWUE is noted only by ca. 3 % (Fig. 3). Literature background (e.g., Saurer et al. 2014; Holtum and Winter 2010) shows that water has been identified as a major issue in a high-CO_2_ world, with the question raised if the effect of elevated CO_2_ on forest vegetation is more of a water issue rather than a carbon issue, emphasizing the strong link between carbon and water cycle. Plants typically react against a decrease in water availability through stomata closure and, although carboxylation rates may also decline under water shortage, leaf conductance is usually affected to a larger extent, originating a reduction in *C*_i_ and a concomitant increase in δ^13^C (Farquhar and Lloyd 1993). Many studies under growth chamber and field conditions have shown that plants developed under water stress (stress induced by low soil water content) and produced leaves with higher δ^13^C (Warren et al. 2001; Ferrio et al. 2003). On the other hand, leaf water availability, the factor that ultimately influences δ^13^C and this availability depends not only on the water input from the soil but also on its physical structure as well as on the hydraulic resistance along the plant xylem (Warren and Adams 2000). Moreover, the rate of evaporation from the leaf also determines stomatal responses that subsequently affect δ^13^C. Indeed, an increase in the leaf-to-air vapor pressure gradient will also cause a reduction in *C*_i_, leading to higher δ^13^C values (Ehleringer 1990). The relationship between δ^13^C and plant water availability is not linear, showing a saturation trend as water availability increases. The reason for that general trend is that the main factor relating δ^13^C with water inputs is stomatal conductance, which is expected to reach its maximum in non-stressed plants (Warren et al. 2001).

## Conclusions

In this study, we have demonstrated that the complementarity of nitrogen and carbon isotope indicators analyzed in needles collected from the areas nearby different plants to provide information on diffuse air pollution is coherent with the investigated region. This approach can provide complementary information to reconstruct and analyze the environmental perturbations. An important highlight of this research is that a complex short-term variation analysis can be helpful in distinguishing isotopic fractionation that is not explainable by climatic conditions; the samples were collected in the same time from all the sampling sites. However, seasonal differences in the isotopic composition of pine needles are observed. Consequently, the separation of the global and local anthropogenic trends for these indicators is based on the comparison stable isotope composition in the needles between the polluted and relatively healthy sampling sites. The visible impact of air pollution on the trees is indicated by the variation of isotopic values in pine needles as a response controlled by physiological mechanisms. Future research on the reconstruction of environmental changes should address the specific question of identifying the mechanisms controlling the short-term changes in isotopic ratio composition. The analyses of tree-ring δ^13^C and δ^15^N variations are promising tools for investigating the nitrogen and carbon deposition to forests. Moreover, regional analyses of needle δ^13^C and δ^15^N variations could enable the mapping of the impact of carbon and nitrogen depositions on ecosystems, assessing the input of pollutants into plant communities. These studies confirm that the physiological responses (stable isotopic fractionation and variation of iWUE at the level of pine needles) were modified as a function of the type of emitters, the space around the emitters (distance and direction from emitters), and local road-effects.
